# A v-transformed copula-based simulation model for lithological classification in an Indian copper deposit

**DOI:** 10.1038/s41598-022-24233-2

**Published:** 2022-12-06

**Authors:** K. Dinda, B. Samanta, D. Chakravarty

**Affiliations:** 1 Advanced Technology Development Centre, Indian Institute of Technology Kharagpur, Kharagpur, 721302 India; 2 Department of Mining Engineering, Indian Institute of Technology Kharagpur, Kharagpur, 721302 India

**Keywords:** Engineering, Mathematics and computing

## Abstract

Copula functions are widely used for modeling multivariate dependence. Since the multivariate data may not necessarily be linear and Gaussian, the copula model is very often brought into the picture for modeling such multivariate phenomena. The lithological classification in spatial domain is a class of problems dealing with categorical variables. A generalized class of copula model is effective for such classification tasks. In this paper, a non-Gaussian copula (v-transformed copula) model has been used for lithotype classification of an Indian copper deposit. Coupling of Markov chain Monte Carlo (MCMC) simulation and copula discriminant function is performed for this purpose. Specifically, four lithotypes, e.g., granite, quartz, basic, and aplite are simulated in the case study deposit. The efficacy of v-transformed copula discriminant function-based simulation is compared with those of Gaussian copula, t copula, and sequential indicator simulations. Finally, the classification accuracy of all the approaches is examined with ground-truth lithological classes obtained from blast hole information. The results show that the v-transformed copula simulation has a relatively higher classification accuracy (76%) than those of Gaussian copula (70%), t copula (69%), and sequential indicator (70%) simulations.

## Introduction

A 3-dimensional geological model representing an ore deposit is constructed by resource geologists^1,2^. The exploration data provides sufficient knowledge through geological field observations, drill-hole logs, geophysical surveys, and assays to assist such deposit modeling. One of the primary tasks in deposit modeling is lithotype mapping. Lithotypes are classified into different categories based on interclass dependencies. However, the following two issues, among others, occur during lithotype modeling in an ore deposit^3^.

- The first one is defining domain layout to minimize misclassifications.
- The second one relates to determining whether or not the geological interpretation is a faithful representation of reality.

Geostatistics offers different estimation techniques, including indicator kriging^4^ and indicator co-kriging^5^ for lithological mapping. But, estimation methods have an inherent deficiency of not predicting uncertainty reliably. Conditional simulation approaches^6,7^ overcome the above shortcomings by creating multiple equal probable scenarios. Many conditional simulation approaches are available in the literature, such as object‐based simulation^7^, sequential indicator simulation^8^, truncated Gaussian simulation^8,9^, simulated annealing^10^, T‐Prog^11^, multiple‐point statistics^12^, pluri‐Gaussian simulation^13^, and genetic as well as pseudo genetic models^14^. Among these approaches, sequential indicator simulation is widely used. However, this approach has certain limitations as highlighted by Emery^15^. Chiles and Delfiner^7^ explain the advantages and disadvantages of other conditional simulation approaches in their book (Geostatistics: Modeling spatial uncertainty). Nevertheless, the following issues occur during the simulation of lithotypes^16^.

- How to estimate spatial correlation of lithotypes efficiently and accurately?
- How to incorporate spatial correlation into lithological classification?
- How to deal with non-Gaussianity of lithotype data?

Auto-correlation within a single lithotype and cross-correlation between lithotypes are usually described by indicator variogram/cross-variogram^17–19^. Li^20^ has proposed an alternative approach, namely transiogram, which can capture spatial auto and interclass dependency structure more flexibly by virtue of its asymmetric and unidirectional irreversible property.

A copula-based approach devised in this study addresses all the above three issues. Notably, the supremacy of copula approaches in multivariate spatial data analysis is advocated by several researchers. For example, these approaches are widely used in frequency analysis of multivariate spatial data^21^, risk analysis at finance^22,23^, analysis of extremes^24^, meteorology and climate research^25,26^, hydrology property^27,28^, soil^29^, and mining applications^30^.

Copula discriminant functions have also been applied for the classification of categorical data. Sathe^31^, Kazianka, and Pilz^32^ have integrated the copula function with the Bayesian classifier. Other examples include the works by several research groups^16,33,34^. The advantages of the copula function are:

- Copula function describes the nonlinear spatial dependence irrespective of marginal distribution.
- It describes full distribution, which is more informative than a variogram-based model.
- For a set of neighboring points, the parameterized copula gives strong dependence (Frechet upper bound) and allows independence for distant points.

Several high-dimensional copula models, such as Archimedean copula^35^ and Fairlie-Gumbel-Morgenstern copula^36^, do not satisfy the above conditions. Gaussian copula^37^ and t copula^38^ fulfill the above conditions. However, these copulas only capture symmetrical dependence. On the other hand, the v-transformed copula introduced by Bardossy and Li^27^ captures the asymmetrical spatial dependence in the upper and lower tails besides fulfilling the above-mentioned conditions. In view of the above, the v-transformed copula discriminant model is used in this study for lithological classification of a copper deposit. The efficacy of model has also been compared with Gaussian copula discriminant, t copula discriminant, and sequential indicator simulation of lithological attributes.

### Study area and data preparation

**Figure 1:**
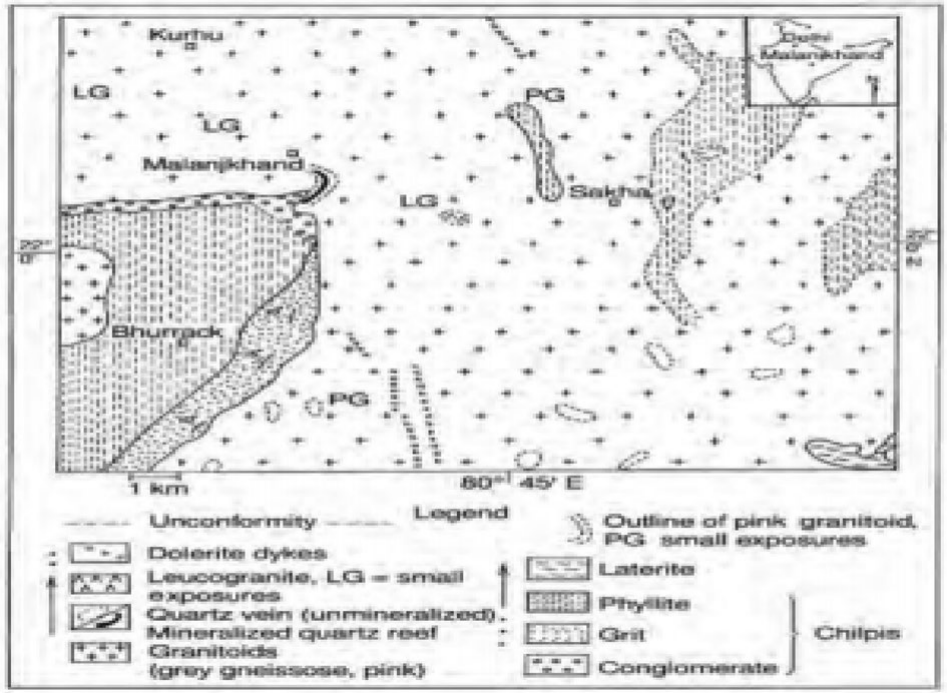
Geological map of the Malanjkhand copper deposit^40^.

**Figure 2:**
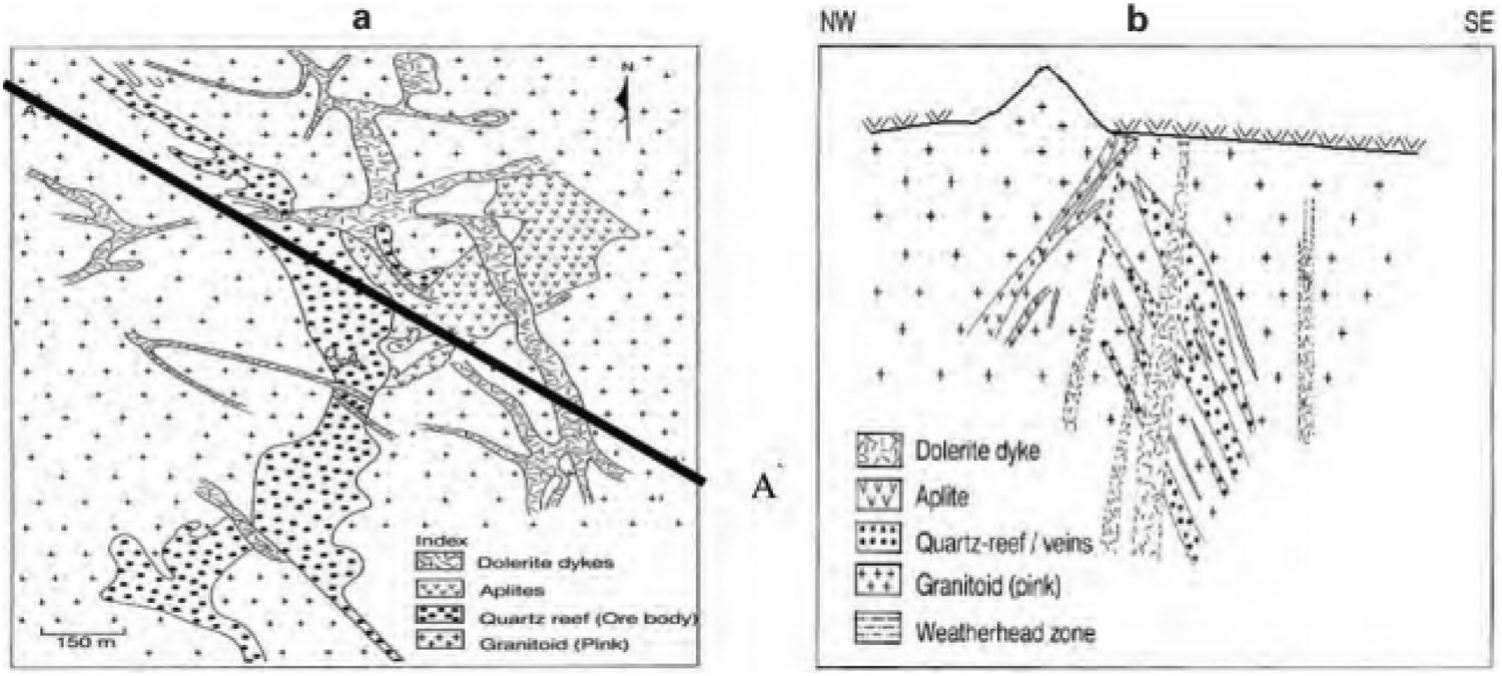
Mine-scale geological map of Malanjkhand copper deposit. (**a**) plain view of different lithotypes (**b**) cross-sectional view of lithotypes along AA′^40^.

**Figure 3:**
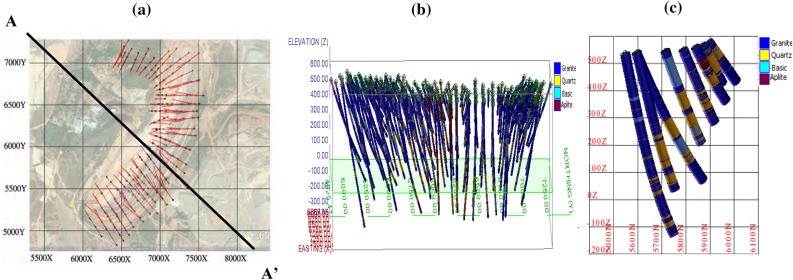
(**a**) Drill hole layout of the deposit, (**b**) down the hole drill hole traces, (**c**) vertical projection of drill holes over a cross-section along AA′. The colour map indicates the different lithotypes.

To begin with the study, exploratory drill hole data is collected from the Malanjkhand copper deposit, which is located at Balaghat district at Madhya Pradesh in India at an altitude of 576 m AMSL (Above Mean Sea Level). The deposit extends over a length of 2200 m along the strike with an orebody width of 600 m. The deposit is currently being mined by an open-pit mining method with a bench height of 12 m and is on the verge of starting underground mining. The geological characteristics of the Malanjkhand copper deposit have been studied by several researchers^39–41^. It reflects that the deposit is predominantly composed of granitoids containing quartz veins, which are interspersed with aplite and dolerite dykes. Copper mineralization occurs mainly in the quartz veins. Figure 1 displays the local geological map of the deposit. A plain and cross-sectional view of the mine-scale geological map are also presented in Fig. 2a,b. The present study is devoted to a specific bench, designated by its mid-bench elevation at 448 m AMSL. Ground-truth blast hole lithological information, which is used in validation exercises and only available from this bench, has endorsed us to select this bench. There are 225 exploratory diamond drill holes made available to us for conducting this study. Interestingly, the drill holes have been drilled mostly inside the copper mineralization veins. As the orebody dips 60°–70° towards the east, most of the drill holes are drilled perpendicular to this direction. The drill holes are placed in an exploration grid of roughly 110 m spacing along the strike and 50–65 m along the dip. Figure 3a presents the drill hole layout in the studied area. Figure 3b displays the drill hole traces exhibiting lithological variation down the hole. Figure 3c also shows a vertical projection of lithology along a selected section AA′.

The raw exploratory drill holes that are supplied to us are recorded in drill core segments. These drill cores are processed by bench-wise compositing method^42^ with a composting length of 12 m, equalling bench height. Since, in this study, a 3-D neighborhood template is defined for simulation at 448 m bench, the drill hole compositing on three consecutive benches: 436 m, 448 m, and 460 m are performed, respectively. These composite samples serve as the primary inputs for the lithological simulation at 448 m bench.

**Figure 4:**
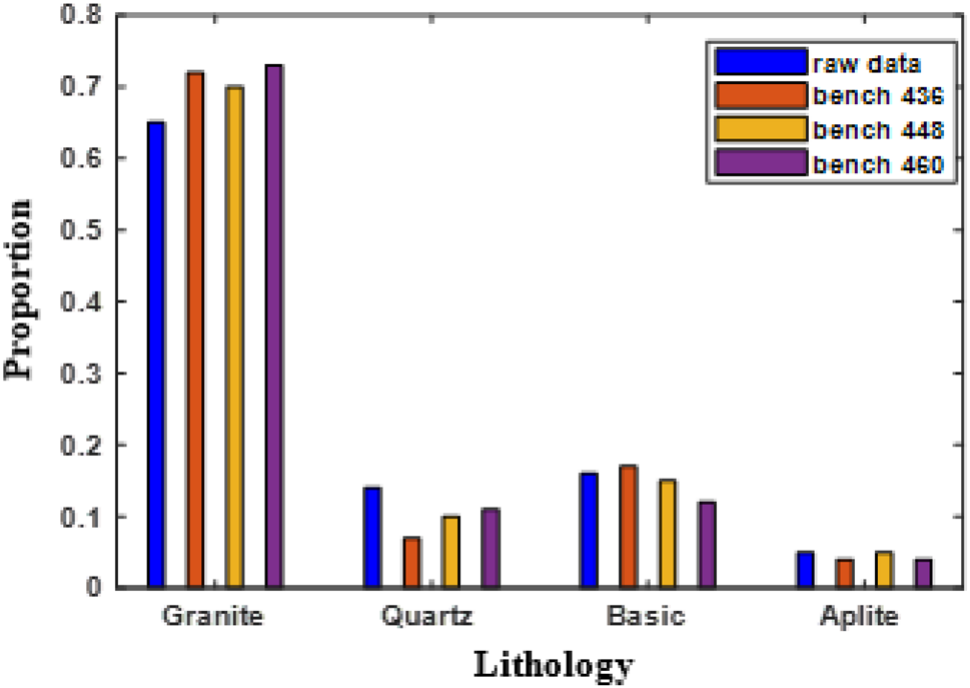
Information on the lithotype proportion computed using raw core and composite samples separately in three consecutive benches: 436 m, 448 m, and 460 m.

Four different lithological units are encountered during exploratory drilling at these benches (shown in Table 1). These are granite, quartz, basic (diorite), and aplite. Figure 4 provides information of the lithotype share computed on the basis of raw core and composite samples for these benches. It can be seen that lithotype share exhibited by raw core and composite samples is more or less comparable. The major share, as per composite sample statistics, is taken by granite (72%), followed by basic (15%) and quartz (9%). The presence of aplite is very scanty (only 4%).

**Table 1:** Descriptive statistics of lithological raw data.

No. of raw data	Granite (%)	Quartz (%)	Basic (%)	Aplite (%)
2655	65	14	16	5

## Material and methods

### Spatial correlation and transiogram construction

The spatial auto-correlation and cross-correlation between lithotypes are captured through transiogram models. The auto and cross transiogram represent transition probabilities from one class to itself and another, respectively, at different lag distances. The transition probability of random variable Z from class *i* to *j* at lag vector ***h*** can be defined by the following equation:

1 $${p}_{ij}\left({\varvec{h}}\right)=prob(Z\left(y+{\varvec{h}}\right)=j|Z\left(y\right)=i)$$

where *i, j* = 1,…., E, E is the number of classes, and *Z(y)* is the random variable defined at location *y*. It can be noted that there exists a theoretical restriction on transiogram (copula as well) to be stationary on a spatial random field. However, the stationarity is considered in almost every cases for practical applications (Goovaerts^43^). One of the reasons is non-availability of enough sample data from geological field, and data are pooled together for estimating spatial correlation structure. Our copula and transiogram models are not free from this theoretical restriction and therefore, an explicit assumption of stationarity is made for transiogram and copula model constructions.

The transiogram computed from sample data points is discontinuous in nature (called experimental). But simulation or estimation of lithotypes at an unknown location requires a continuous transiogram model (called theoretical transiogram). Usually, a parametric or interpolation technique is used to develop a continuous model. However, the parametric model, such as spherical, Gaussian, or elliptical, which is commonly used for semi-variogram modeling, may not be appropriate for transiogram modeling^44,45^. Therefore, an interpolation method is used. In this study, a nonlinear interpolation method, namely piecewise cubic Hermite interpolation^46^ has been used to model the continuous transiogram. A brief discussion of this interpolation method is provided below.

Let us now consider $h_{k}$ and $h_{k + 1}$ ($h_{k + 1} > h_{k}$) are two consecutive lag distances (norms of lag vectors) for a given direction. The $p_{ij} (h_{k} )$ and $p_{ij} (h_{k + 1} )$ are two transition probability values calculated from sample data at lag distances $h_{k}$ and $h_{k + 1}$, respectively. The monotone piecewise cubic Hermite interpolation equation for calculating the transition probability value $\mathop {p_{ij} }\limits^{ \wedge } (h)$ at distance $h$ can be expressed as:

2 $$\begin{aligned} \mathop p\limits^{ \wedge }_{ij} (h) & = \frac{{p_{ij} (h_{k + 1} ) \times (3ts^{2} - 2s^{3} ) + p_{ij} (h_{k} ) \times (t^{3} - 3ts^{2} + 2s^{3} )}}{{t^{3} }} \hfill \\ & \quad + \frac{{d_{ij} (h_{k + 1} ) \times (s^{2} (s - t)) + d_{ij} (h_{k} ) \times (s(s - t)^{2} )}}{{t^{2} }} \hfill \\ \end{aligned}$$

where, $t = h_{k + 1} - h_{k}$, $s = h - h_{k}$, $d_{ij} (h_{k} )$ and $d_{ij} (h_{k + 1} )$ are the slopes of the interpolant at lag distances $h_{k}$ and $h_{k + 1}$, respectively.

**Figure 5:**
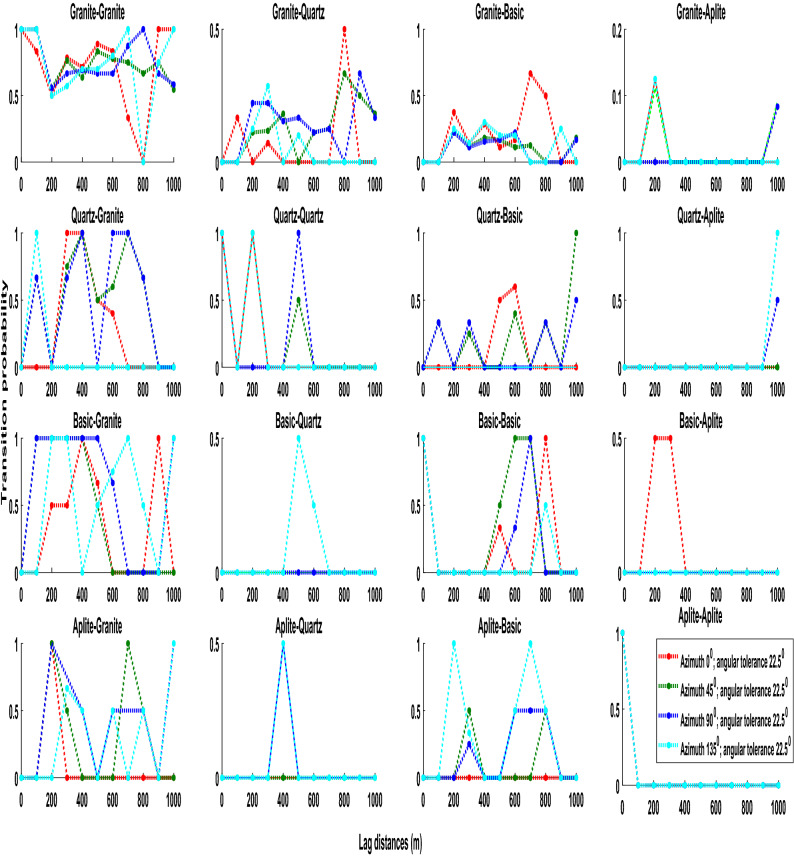
Four directional auto/cross-transiograms at different lag distances.

The transiogram study is initiated with a direction transiogram modeling along four different azimuth directions such as 0°, 45°, 90°, 135° with an angular tolerance of 22.5°. Figure 5 presents the directional auto-transiogram and cross-transiogram. It appears that transiograms are very erratic. No systematic directional transiogram pattern is deciphered by this investigative study. As a follow-up process, omni-directional transiogram modeling is also carried out.

**Figure 6:**
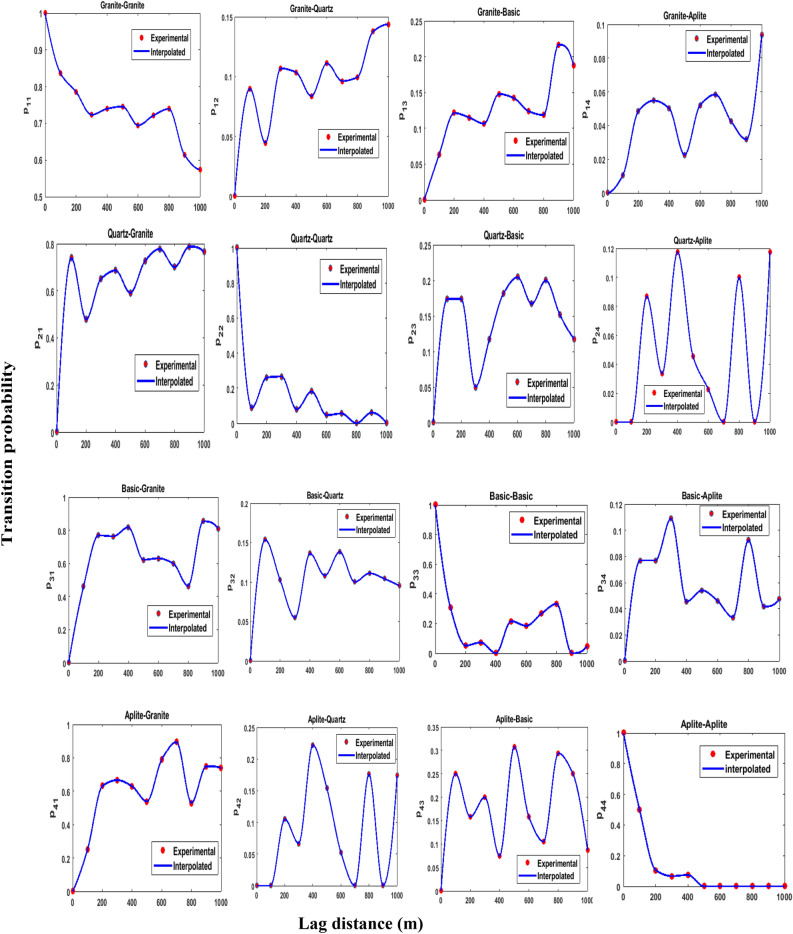
Experimental auto/cross transiograms and theoretical transiograms based on nonlinear interpolation for four lithotypes (granite; quartz; basic; aplite) at different lag distances.

Figure 6 presents the omni-directional auto and cross transiograms for four lithological classes. The dotted points (red color) in the graph represent experimental transition probabilities at different lags. They look relatively better than the directional transiograms. Hence, it is decided to use an omni-directional transiogram for later part of directional transiogram study. It is important to stress upon the fact that some of these transiograms are complex in shape. This might be due to a lack of regular spatial structure confounded with few data points present in certain lithological classes. Consequently, Hermite polynomial interpolation is used to fit the theoretical transiogram models (solid blue line).

### Simulation for lithological classes

**Figure 7:**
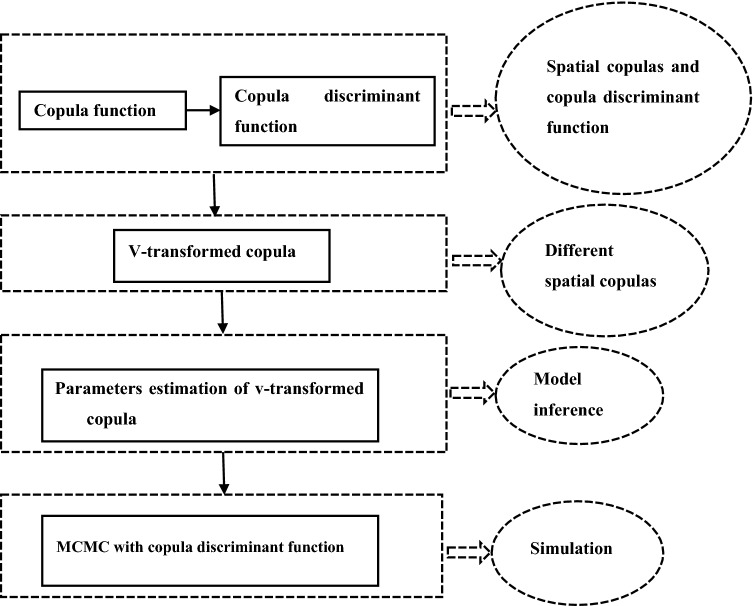
Flowchart of the proposed model and method for simulation.

This section presents the theoretical foundation of copula discriminant function and Markov Chain Monte Carlo (MCMC) simulation. Figure 7 describes the flowchart of the simulation procedure adopted here.

#### Spatial copulas and copula discriminant function

Copula describes dependencies among variables without being influenced by marginal distribution. According to Silva and Lopes^47^, an *N*-dimensional copula is a distribution function defined on a unit hypercube [0,1]^*N*^. It connects the joint distributions with their univariate marginal distributions.

3 $$C(F(p_{1} ), \ldots ,F(p_{N} )) = F(p_{1} , \ldots ,p_{N} )$$

where, $F(p_{1} ),\ldots,F(p_{N} )$ be marginal cumulative distribution functions (CDF), $F(p_{1} ,\ldots,p_{N} )$ be *N-*dimensional joint distribution function of random vectors ***P***, $C:[0,1] \times \cdots \times [0,1] \,$ be a copula function of the transformed random vectors ${{\varvec{U}}}_{i}=F({p}_{i}), \, {\text{i}}=1,\ldots , \, {\text{N}} \,$(Schweizer and Sklar^48^).

Sklar^49^ proposes the converse of Eq. (3). This means an *N*-dimensional joint distribution can be expressed in the form of a copula function.

4 $$F(p_{1} , \ldots ,p_{N} ) = C(F(p_{1} ), \ldots ,F(p_{N} ))$$

The copula function C in Eq. (4) is unique. When the marginal distribution is continuous, it can be represented by the following equation (Nelsen^50^).

5 $$\begin{aligned} c(u_{1} ,\ldots,u_{N} ) & = \frac{{\partial^{N} c(u_{1} ,\ldots,u_{N} )}}{{\partial u_{1} ,\ldots,\partial u_{N} }} \hfill \\ & = \frac{{f(p_{1} ,\ldots,p_{N} )}}{{f_{1} (p_{1} ), \ldots ,f_{N} (p_{N} )}} \hfill \\ \end{aligned}$$

where *c* is the copula density function, $f(p_{1} , \ldots ,p_{N} )$ is the joint density function and $f_{1} , \ldots, f_{N}$ are marginal density functions (PDF) of corresponding marginal CDFs $F_{1} ,\ldots,F_{N}$.

For lithological classification**,** let assume $\Omega = \left\{ {\omega_{1} ,\ldots,\omega_{E} } \right\}$ be *E* lithological classes observed in the geological domain. Furthermore, each sample point $z(y_{e} )$ at a location $y_{e}$ from *e*th class is surrounded by *n* neighboring locations $y_{1} ,\ldots,y_{n}$ with samples $z(y_{1} ),\ldots,z(y_{n} )$, respectively. If individual pair-wise spatial interaction between $z(y_{e} )$ and each of its spatial neighbors is represented by transition probability vector ${{\varvec{T}}}_{e}=\left\{{T}_{1e},\ldots,{T}_{ne}\right\}$, then the task of classification is to assign each vector ***T***_*e*_ to a lithological class $\omega_{e}$. A Bayesian classifier assigns *T* to *e*th class if $g({\varvec{T}}_{e} ) \succ g({\varvec{T}}_{f} ) \, \forall f \ne e$.

Where $g:[0,1]^{n} \to [0,1]$ is the copula discriminant function, which is defined by:

6 $$g({\varvec{T}}_{e} ) = \ln P(\omega_{e} ) + \ln c\left( {F_{1} (T_{1e} \left| {\omega_{e} ),\ldots,F_{n} (T_{ne} \left| {\omega_{e} )} \right.} \right.\left| {\omega_{e} } \right.} \right) + \sum\limits_{i = 1}^{n} {\ln f_{i} (T_{ie} \left| {\omega_{e} )} \right.}$$

For convenience to the readers, detailed derivation of the copula based discriminant function is presented in Appendix A.

From Eq. (6), it is noticed that the copula discriminant function has three parts: the prior probability $P(\omega_{e} )$, the conditional copula density $c\left(\cdot{\left| {\omega_{e} } \right.} \right)$ and the conditional marginal densities $f_{1} \left(\cdot {\left| {\omega_{e} } \right.} \right),\ldots,f_{n} \left(\cdot {\left| {\omega_{e} } \right.} \right)$. These three components are separated, and all the discriminant information in the model is embedded in the copula density. Without prior probability, the conditional copula density and marginal density are related to transition probabilities. Therefore, this is called a spatial copula discriminant function, which differs from the traditional copula^33^. The Gaussian, t, or v-transformed copula density can be used in Eq. (6) to get the respective spatial discriminant function.

#### V-transformed Copula

In literature, different families of copulas are found. Copulas are distinguished by their parameters, which capture the diversified structures of dependencies. Copula family is differentiated by its upper tail or lower tail distribution, which describes the significance of dependence at the upper or lower quantile. The v-transformed copula is quite similar to the Gaussian copula, but the former has two additional parameters, one is the scale parameter (*m*), and another is the shape parameter (*k*). It is worth to mention that the shape of conditional and unconditional copulas is assumed to be the same. This is rather a strong assumption. However, many of the earlier investigations of the v-transformed copula model such in hydrology (Bardossy and Li^27^; Li^28^); rainfall prediction (Aghakouchak et al.^51^); hydraulic conductivity estimation (Haslauer et al.^52^; Guthke^53^); resource estimation of mineral deposits (Dinda and Samanta^30^) have used this model in the above setting. Therefore, the above assumption is explicitly adopted in this study.

The v-transformed copula is a non-monotonic transformation of multivariate Gaussian copula (Bardossy and Li^27^). This transformation captures a wide variety of asymmetrical spatial dependence structures in multivariate distribution. The non-monotonic transformation is defined as:

7 $${\varvec{Y}}_{i} = \left\{ {\begin{array}{ll} { k({\varvec{G}}_{i} - m)} & \quad { if \; {\varvec{G}}_{i} \ge m } \\ { m - {\varvec{G}}_{i}} & \quad { if \; {\varvec{G}}_{i} \prec m } \\ \end{array} } \right.$$

where ${\varvec{G}}_{i}$ is an *N-*dimensional standard normal distribution with a mean vector zero and correlation matrix ${\varvec{\rho}}$. The parameters *k* and *m* maintain the structure of the distribution.

According to Bardossy and Li^27^, the marginal distribution of ***Y***_***i***_ can be expressed as:

8 $$F(y) = \Phi \left (\frac{y}{k} + m \right) - \Phi ( - y + m)$$

Also, the marginal density is

9 $$f(y) = \frac{1}{k}\phi \left (\frac{y}{k} + m \right) + \phi ( - y + m)$$

where $\Phi$ and $\phi$ is the standard Gaussian CDF and density function.

The v-transformed copula density function can be described as:

10 $$c_{{m,k,\varvec{\rho }}} (u_{1} , \ldots ,u_{N} ) = \frac{{(2\Pi )^{{ - \left( {N/2} \right)}} \left| \varvec{\rho } \right|^{{ - \left( {1/2} \right)}} \sum\nolimits_{{i = 0}}^{{2^{N} - 1}} {\frac{1}{{k^{{N - \sum\nolimits_{{j = 0}}^{{N - 1}} {a_{j} } }} }}\exp \left( { - \frac{1}{2}(\varvec{\xi }_{i} + \varvec{m})^{T} \varvec{\rho }^{{ - 1}} (\varvec{\xi }_{i} + \varvec{m})} \right)} }}{{\prod\nolimits_{{i = 1}}^{N} {\left( {\frac{1}{k}\phi \left( {\frac{{y_{i} }}{k} + m} \right) + \phi ( - y_{i} + m)} \right)} }}$$

where $${\varvec{\xi}}_{i}^{T} = \left( {b( - 1)^{{a_{1} }} y_{1} \ldots, b( - 1)^{{a_{N} }} y_{N} } \right)$$,

$$a_{j} = 0{\text{ or 1}}$$ and $$i = \sum\nolimits_{j = 0}^{N - 1} {a_{j} 2^{j} }$$

11 $$b = \left\{ {\begin{array}{ll} { - 1} & \quad {{\text{if}} \; ( - 1)^{{a_{j} }} = - 1} \\ {\frac{1}{k}} & \quad {{\text{if}} \; ( - 1)^{{a_{j} }} = 1} \\ \end{array} } \right.$$

The parameters *k, m,* and ${\varvec{\rho}}$ play a significant role in capturing different spatial dependence structures.

**Figure 8:**
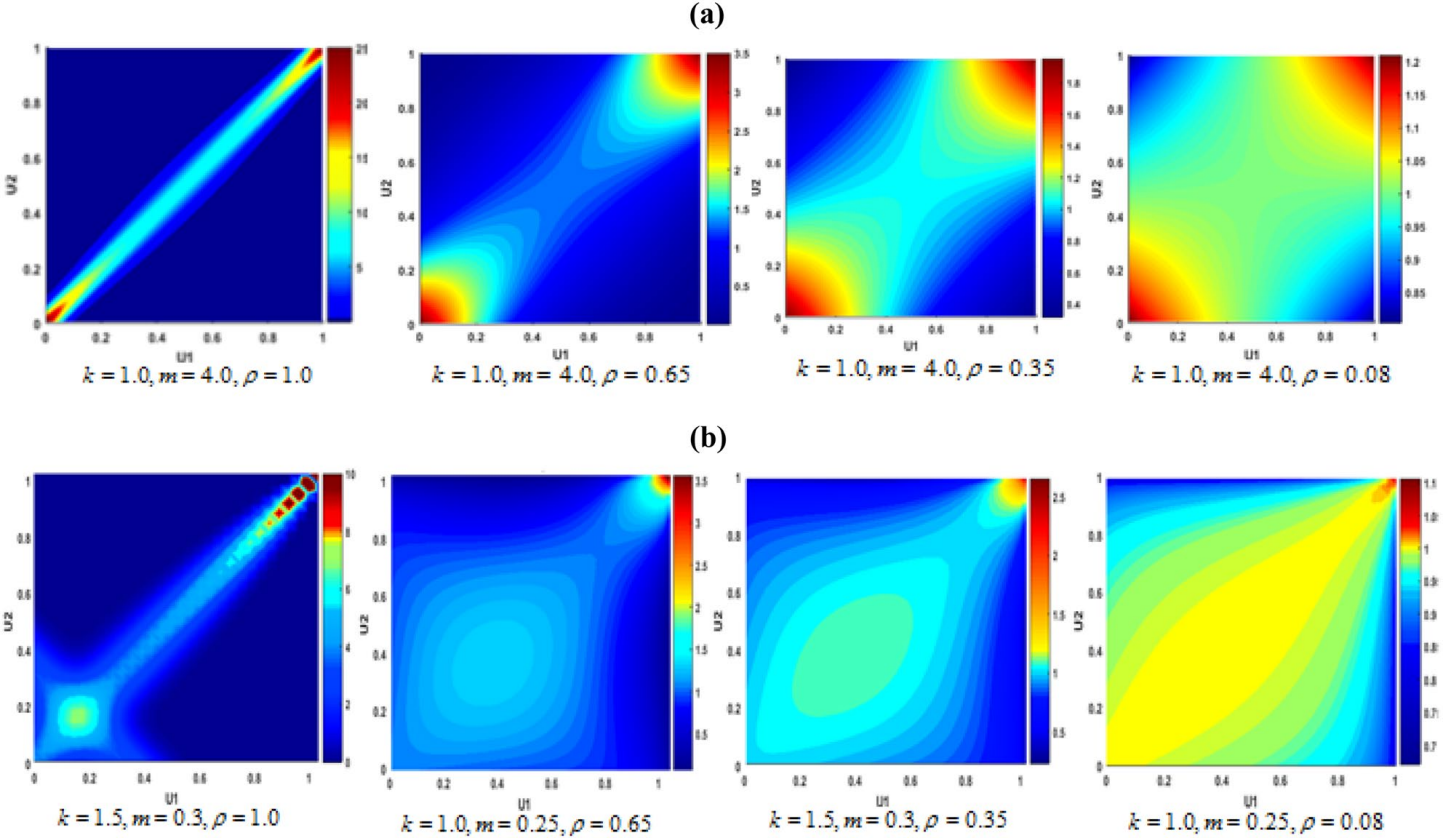
(**a**) Bivariate Gaussian copula densities with different *ρ*, (**b**) Bivariate v-transformed copula densities with different *ρ*, *m* and *k*.

For example, when *k* = 1 and *m* = 4, the v-transformed copula converges to Gaussian copula. However, with decreasing $\rho$, the bivariate distribution patterns change. This is shown in Fig. 8a. For other values of *k*, *m*, and $\rho$ different bivariate patterns look like as in Fig. 8b.

#### Statistical inference method

The class conditional density $f({\varvec{T}}/\omega_{e} )$ for a feature vector **T** is defined by v-transformed copula model. In order to construct this density function, the parameters of the copula model has to be estimated. This is done by extracting training samples from known observations of drill hole locations belonging to a particular class in the geological domain. It may be noted that each input pattern of training samples is an instance of an n-dimensional random vector of transition probability **T** constituted by n neighborhood observations. Therefore, sufficient numbers of training samples are generated for each class. These training samples are used to compute the values of transition probability using auto and cross transiograms. A maximum likelihood method is then used for parameter estimation of the copula class conditional density functions. The procedure of parameter estimation is provided below:

Suppose *O*_*e*_ number of training patterns are used for *e*th class and the input vector of transition probability ${{\varvec{T}}}_{{\varvec{e}}}=\left\{{{\varvec{T}}}_{1e}^{(p)},......,{{\varvec{T}}}_{ne}^{(p)}\right\}\text{, }p=1,......,{O}_{e}$ is constructed using the above training patterns in this exercise. The general form of log-likelihood function for estimating the parameters ${\varvec{\theta}}_{e} = (m_{e} ,k_{e,} {\varvec{\rho}}_{e} )$ can be represented as:

12 $$l({\varvec{\theta}}_{e} ) = \sum\limits_{p = 1}^{{O_{e} }} {\ln c(F_{1} (t_{1e}^{(p)} ;m_{e} ,k_{e} } ), \ldots, F_{n} (t_{ne}^{(p)} ;m_{e} ,k_{e} );{\varvec{\rho}}_{e} ) + \sum\limits_{p = 1}^{{O_{e} }} {\sum\limits_{i = 1}^{n} {\ln f_{p} (t_{ie}^{(p)} ;m_{e} ,k_{e} )} }$$

where $F_{e}$ and $f_{e}$ be the marginal CDF and PDF, respectively.

The maximum likelihood estimation (MLE) of ${\varvec{\theta}}_{e}$ can be expressed as

$$\mathop {\varvec{\theta}}\limits^{ \wedge }_{e} = \mathop {\arg \max }\limits_{{{\varvec{\theta}}_{e} \in \Theta }} l({\varvec{\theta}}_{e} )$$

where $\Theta$ is parametric space.

The log-likelihood function for v-transformed copula is

13 $$\begin{aligned} l({\varvec{\theta}}_{e} ) & = \ln (c_{{m_{e} ,k_{e} ,{\varvec{\rho}}_{e} }} (\mathop {u_{1} }\limits^{(p)} ,\ldots,\mathop {u_{n} }\limits^{(p)} )), \, p = 1,\ldots,O_{e} \hfill \\ & = - \frac{{O_{e} }}{2}\ln \left| {\mathop {{\varvec{\rho}}^{ - 1} }\limits_{e} } \right| + \sum\limits_{p = 1}^{{O_{e} }} {\ln (\sum\limits_{i = 0}^{{2^{n} - 1}} {\frac{1}{{k_{e}^{{n - \sum \nolimits_{jj = 0}^{n - 1} {a_{jj} } }} }}} } \exp ( - \frac{1}{2}({\varvec{\xi}}_{i}^{(p)} + {\varvec{m}}_{e} )^{T} {\varvec{\rho}}_{e}^{ - 1} ({\varvec{\xi}}_{i}^{(p)} + {\varvec{m}}_{e} ))) \hfill \\ & \quad + \frac{1}{2}\sum\limits_{p = 1}^{{O_{e} }} {\sum\limits_{i = 1}^{n} {\ln f_{p} (t_{ie}^{(p)} ;m_{e} ,k_{e} )} } \hfill \\ \, \hfill \\ \end{aligned}$$

where $${\varvec{\theta}}_{e} = \left\{ {\left( {m_{e} ,k_{e} ,{\varvec{\rho}}_{e} } \right)\left| {m_{e} \in ( - \infty ,\infty );k_{e} \in (0,\infty );{\varvec{\rho}}_{e} \in [ - 1,1]^{n \times n} } \right.} \right\}$$.

When the number of neighbours increases, the copula parameters are challenging to estimate through the maximum likelihood function. Joe and Xu^54^ have proposed a two-stage estimation procedure. The efficiency of the two-stage estimation method for the copula-based model is discussed by Yan^55^; Ko and Hjort^56^. The same approach has been employed in this study and is described in Appendix B.

For the current study, the optimal parameters *m*, *k*, and correlation matrix ${\varvec{\rho}},$ as found from the data, are listed in Tables 2 and 3.

**Table 2:** The optimal parameters (*m, k)* of v-transformed copula for four lithotypes.

	Granite	Quartz	Basic	Aplite
*m* (%)	0.29	0.24	0.25	0.20
*k* (%)	1.42	1.11	1.20	1.04

**Table 3:**
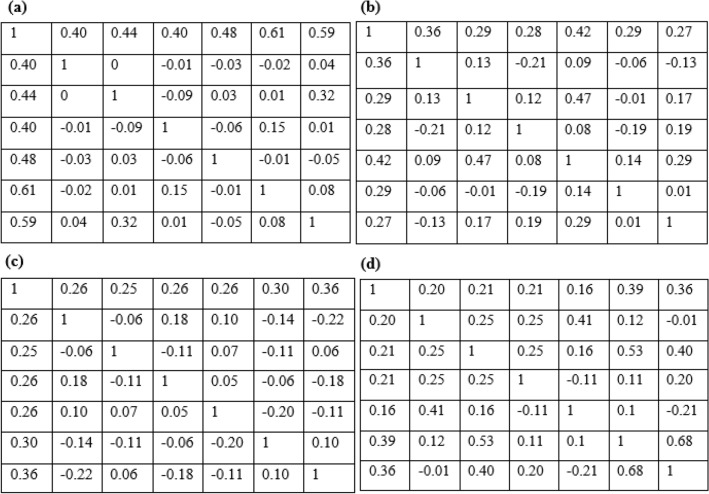
The correlation matrix of v-transformed copula for four lithotypes: (a) granite; (b) quartz; (c) basic; (d) aplite.

#### Markov chain Monte Carlo simulation

Markov chain Monte Carlo simulation (MCMC) is used with Gibbs sampling^57^ for lithological simulation. The detailed procedure is explained in Appendix C. For Gibbs sampling, the six-neighborhood template as presented in Figure S1, is employed. Usually, full conditional data set is used to generate the local conditional distribution of Gibbs sampling. However, in our case, it is only practical to use limited neighborhood as calculation of v-transformed copula density requires a summation of 2^n^ terms (n = the size of neighborhood). The computational time and memory size grow exponentially with an increased neighborhood size. In this respect, a study conducted by Emery et al.^58^ suggests that trimming of full neighborhood by local neighborhood using kriging is not a convenient approach. Particularly, it runs into convergence problems as well as non-reproduction of covariance function. On the other hand, several techniques also available in the literature (Besag^59^; Besag and Kooperberg^60^; Gilks et al.^61^; Cares^62^; Reu and Tjemeland^63^; and Li^64^) suggest that a Gaussian Markov Random Function (GMRF) based approach with an optimized parameter of limited template can guaranteed the reproduction of covariance. Our approach thus relates to the latter approach with optimization of GMRF parameters and then using these parameters to draw samples from conditional distribution. The convergence has been achieved after 500 iterations.

**Figure 9:**
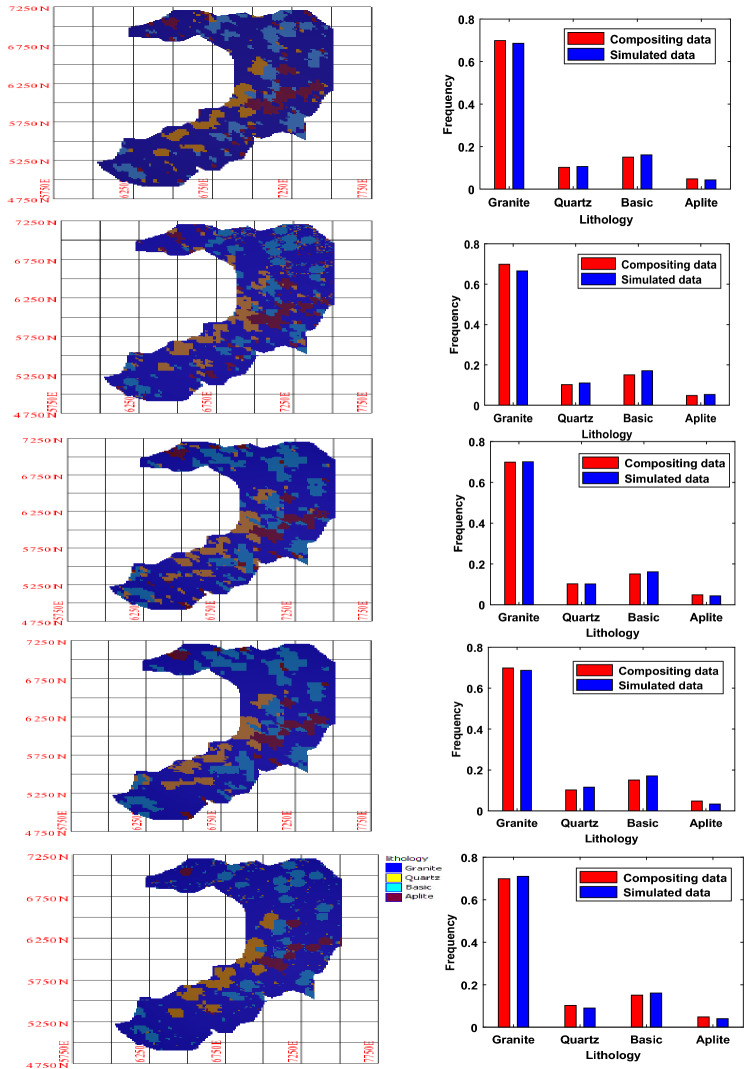
Five simulated maps and bar charts among 50 simulated realizations of v-transformed copula-based model for bench 448 m AMSL.

**Figure 10:**
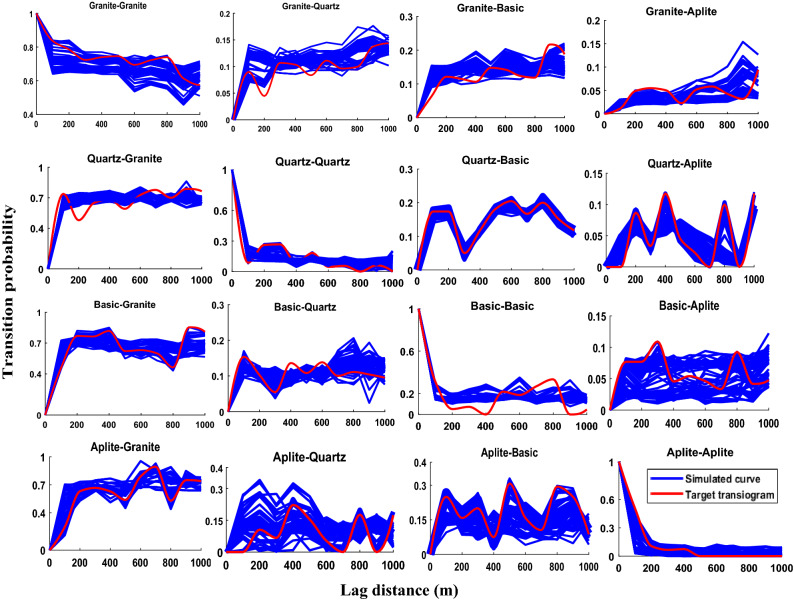
Transiograms of 50 simulated realizations and target transiogram of four lithotypes.

The simulation has been performed for 448 m bench at the grid intervals of 12 m × 12 m with 10,944 grid points. Overall, 50 simulated realizations are analyzed for lithological interpretation. Figure 9 exhibits the example maps along with bar charts of five realizations produced by the v-transformed copula simulation. It can be seen that univariate statistics (bar chart), as well as transiograms, are well reproduced by the simulations. Transiogram curves of 50 realizations superimposed with data transiogram are also shown in Fig. 10.

## Validation exercise

**Figure 11:**
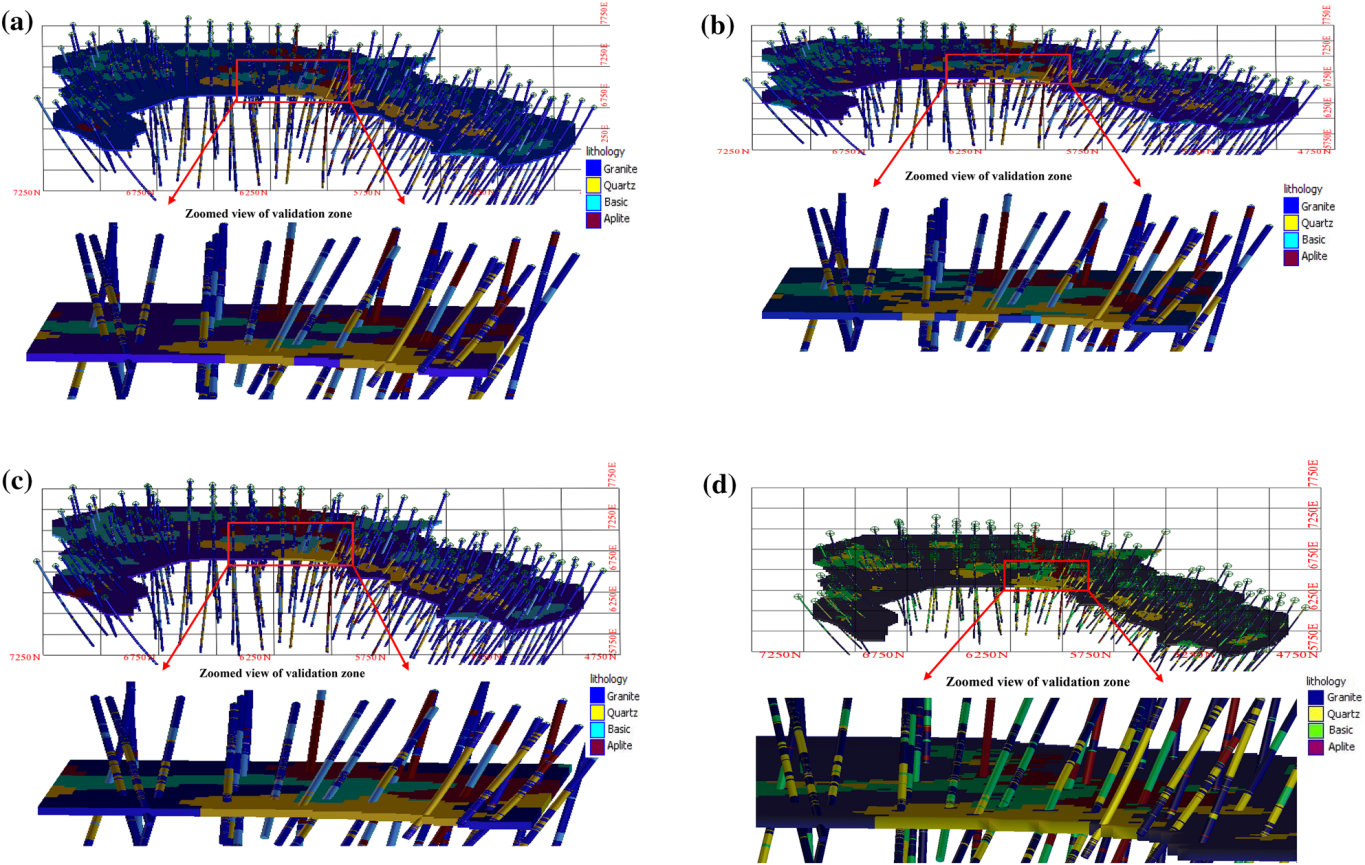
(**a**): Visual examination of MPL maps using Gaussian copula-based simulation. (**b**) Visual examination of MPL maps using t copula-based simulation. (**c**) Visual examination of MPL maps using v-transformed copula-based simulation. (**d**) Visual examination of MPL maps using sequential indicator simulation.

**Figure 12:**
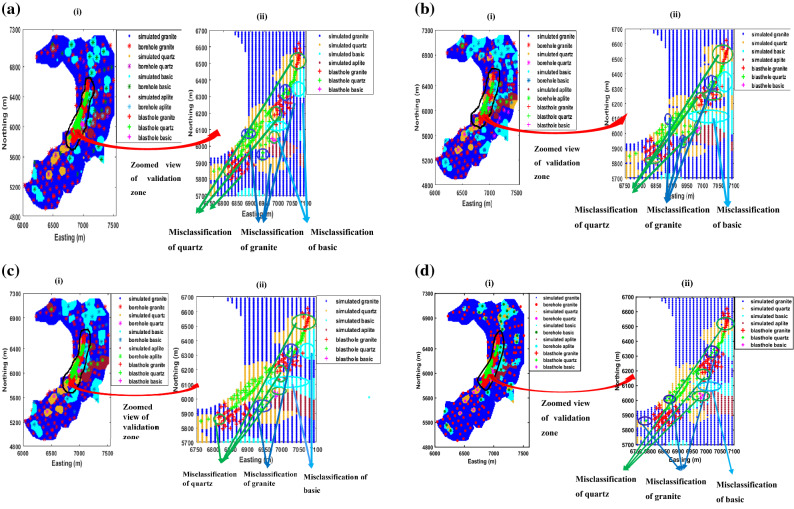
(**a**) MPL maps using Gaussian copula-based simulation. Locations of (i) simulated values, drill holes and blast-holes of bench at 448 m AMSL, (ii) Zoomed view of simulated values and blast-holes of the validation zone of Gaussian copula. (**b**) MPL maps using t copula-based simulation. Locations of (i) simulated values, drill holes and blast-holes of bench at 448 m AMSL, (ii) Zoomed view of simulated values and blast-holes of the validation zone of t copula. (**c**) MPL maps using v-transformed copula-based simulation. Locations of (i) simulated values, drill holes and blast-holes of bench at 448 m AMSL, (ii) Zoomed view of simulated values and blast-holes of the validation zone of v-transformed copula. (**d**) MPL maps using sequential indicator simulation. Locations of (i) simulated values, drill holes and blast-holes of bench at 448 m AMSL, (ii) Zoomed view of simulated values and blast-holes of the validation zone of sequential indicator simulation.

Since the ground-truth lithological information from blast holes is available, a validation exercise is carried out to examine further quality checks of the simulation. For this purpose, a single Most Probable Lithological (MPL) map (for each grid point, the particular lithological class which occurs most of the times out of 50 simulations) is prepared. The MPL values are compared with the ground-truth blast hole values observed nearest to the corresponding grid point locations. Additionally, the outputs of the v-transformed copula simulation are compared with Gaussian copula, t copula and sequential indicator simulation to examine how the former model fairs against later three models. Consequently, 50 realizations from Gaussian copula, t copula, and sequential indicator simulation are generated, and MPL maps are created from them. For brevity, individual simulations, their univariate statistic, and transiograms of Gaussian copula, t copula, and sequential indicator simulation have not been provided in the manuscript. Altogether 172 ground-truth blast hole lithological values are available to inspect this accuracy check. Prior to exercising the ground-truth validation, the MPL maps of these models are also compared visually with the composite lithological values of drill hole data. Figure 11a–d presents the visualization of drill holes with MPL maps based on (i) Gaussian copula, (ii) t copula, (iii) v-transformed copula, and (iv) sequential indicator simulation. The harmony between lithotypes sequence in the drill holes and MPL values of lithotypes for four models are clearly depicted in the above figure. It appears that all the simulations are reasonably confirmative to real scenario. The locations of blast holes along with MPL maps of (i) Gaussian copula, (ii) t copula, (iii) v-transformed copula, and (iv) sequential indicator simulation superimposed with drill hole locations, are also shown in Fig. 12a–d. In this figure, zoomed view of the validation zone is also presented to have a better grasp of comparative analysis. The classification accuracy of three copula models and sequential indicator simulation is numerically computed and presented in a confusion matrix, as shown in Table 4. From this table, it can be seen that the classification accuracy of the v-transformed copula, Gaussian copula, t copula, and sequential indicator simulation are 76%, 70%, 69%, and 70%, respectively.

**Table 4:**
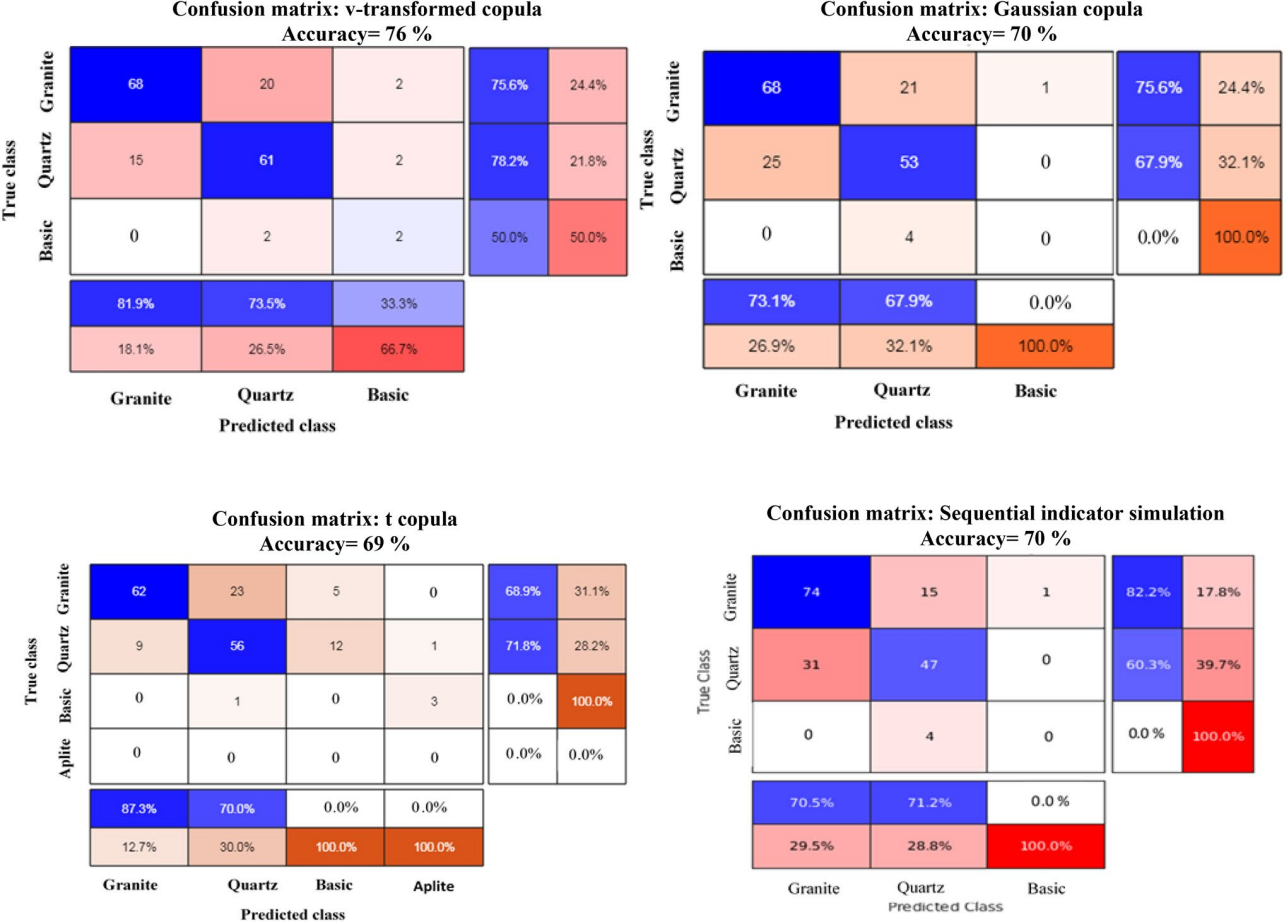
Percentage of classification accuracy of v-transformed copula, Gaussian copula, t copula, and sequential indicator simulations for four lithotypes.

## Conclusion

In this study, a Markov chain Monte Carlo simulation integrated with a v-transformed copula discriminant function is adopted to classify lithotypes in an Indian copper deposit. The outputs of the v-transformed copula simulation map have been compared with those of Gaussian copula, t copula, and sequential indicator simulation. A validation exercise has also been conducted for the most probable lithological maps produced from simulated realizations of the above four approaches with a use of ground-truth blast hole lithological information. The validation exercise reveals that the v-transformed copula discriminant function provides relatively better classification accuracy (76%) than that of Gaussian copula discriminant function (70%), t copula discriminant function (69%), and sequential indicator simulation (70%). However, it is also true that the reported classification accuracy is based on the limited test data set available. Therefore, our interpretation due to validation exercise is confined to this limited data set only.

## Supplementary Information

Supplementary Information.

## Data Availability

The data that supports the findings of this study are available within the article. The data sharing is not applicable to this article as no new data were created in this study.
