# Supplementary material for: A v-transformed copula-based simulation model for lithological classification in an Indian copper deposit
Source: Sci Rep. 2022 Dec 6;12:21055. doi: 10.1038/s41598-022-24233-2 (PMC9726882; doi:10.1038/s41598-022-24233-2)
Supplement: Supplementary file 1 — Supplementary Information. [file 41598_2022_24233_MOESM1_ESM.docx]

**A non-Gaussian copula-based approach for lithological simulation in an Indian copper deposit**

**K. Dinda^1*^, B. Samanta^2*^ and D. Chakravarty^2^**

*^1^ Advanced Technology Development Centre, Indian Institute of Technology Kharagpur, India-721302.*

*^2^Department of Mining Engineering, Indian Institute of Technology Kharagpur, India-721302.*

**APPENDIX A**

This Appendix introduces a discriminant function using copula function. The discriminant function for *e*^th^ class can be written using Basyes theorem:

(A.1)

Where, $P(\omega_{e}\left|T_{1e},.......,T_{ne})\right.$ is posterior probability of lithological class $\omega_{e}$. The prior probability $P(\omega_{e}),\text{ }e=1,.......,E$ can be empirically calculated through the fractions of lithological data belonging to each class. $P(T_{1e},......,T_{ne}\left|\omega_{e}\right.)$ is the conditional probability density function of $\omega_{e}$. Therefore, $P(T_{1e},......,T_{ne}\left|\omega_{e}\right.)$ can be represented as $f(T_{1e},......,T_{ne}\left|\omega_{e}\right.)$. Where $P(T_{1e},......,T_{ne})$ is a constant, and $P(T_{1e},......,T_{ne})=\sum_{e=1}^{E} P(\omega_{e})f(T_{1e},......,T_{ne}\left|\omega_{e})\right.$. Then Eq. (A.1) can be expressed as:

(A.2)

The discriminant function is unchanged if is replaced by , is a monotonically increasing function^I^. Very often, *G* is chosen as natural logarithm transformation and the discriminant function can be written as:

(A.3)

As per Eq. (5), the likelihood function can be expressed in terms of copula density function by the following expression.

$f(T_{1e},......,T_{ne}\left|\omega_{e})\right.=c\left(F_{1}(T_{1e}\left|\omega_{e}),...,F_{n}(T_{ne}\left|\omega_{e})\left|\omega_{e}\right.\right.\right.\right)\prod_{i=1}^{n} f_{i}(T_{ie}\left|\omega_{e})\right.$ (A.4)

Therefore, the discriminant function in Eq. (A.3) can be expressed as:

(A.5)

**APPENDIX B**

Two-stage procedure for copula parameter estimation.

Stage 1:

At first, the parameters $\beta_{e}=(m_{e},k_{e})$ for e^th^ class copula density are optimized through MLE

procedure. Considering stationarity, all the training samples are pooled in, and the log-likelihood

function $l(\beta_{e})$is constructed as:

$$l(\beta_{e})=\sum_{p=1}^{O_{e}} \sum_{i=1}^{n} \ln f_{p}(t_{ie}^{(p)};m_{e},k_{e})$$

A gradient-based non-linear optimization function available in the MATLAB optimization toolbox

(Coleman et al., 1999^II^) is then used to estimate the parameters.

Stage 2:

Conditioned on the estimated $\beta^{\wedge} _{e}$ parameter vector, the correlation parameter matrix of the copula

($\rho_{e}$) is then optimized through the following log-likelihood function.

The standard optimization method cannot be applied to maximize the log-likelihood function, as the number of dimensions of the correlation matrix is high. Therefore, in this paper, an efficient procedure introduced by Hernández et al.^III^ is used to estimate the correlation parameters of the copula. The steps of this algorithm, which employes the inverse gradient decent method, are as follows:

Step1: Estimate the marginal distribution function $\mathop{F}\limits^{\wedge} _{e}$ based on each random variable $T_{ie}$, *i*=1,….,*n*

using the observed transition probability value $\mathop{t_{ie}}\limits^{(O_{e})}$ for *e*^th^ class.

Step2: Calculate for *i=*1*,..…,n; p=*1*,……,O_e._*

Step 3: Transform $u_{ie}^{(p)}$ using the inverse univariate distribution $v_{ie}^{(p)}=F^{-1}(u_{ie}^{(p)})$, $F^{-1}$ is the inverse of

the v-transformed copula CDF given in Eq. (8).

Step 4: Choose an initial value of a covariance matrix $\Sigma_{e(0)}$.

Step 5: Project the covariance matrix space to the correlation matrix space by the following

expression:

$$\rho_{e(0)}=B_{(0)}\Sigma_{e(0)}B_{(0)}$$

Where, $(B_{(0)})_{jk}=\frac{\delta_{jk}}{\sqrt{(\sum_{(0)})_{jj}}}$, and $\delta_{jk}$ is the Kronecker delta is defined as:

$$\delta_{jk}=\text{ }\left\{\begin{matrix} \text{1 if }j=k \\ \text{0 if }j\neq k \end{matrix}\right.$$

The inverse gradient direction can be computed by following expression:

$d(\rho_{e})$ is the derivative matrix of the log-likelihood function (Eq. 13), i.e., $d(\rho_{e})=\frac{\partial l}{\partial\rho_{e}^{-1}}$ $d(\rho_{e})=\frac{O_{e}}{2}\rho_{e}-\frac{1}{2}\sum_{p=1}^{O_{e}} \sum_{i=0}^{2^{n}-1} (\xi_{i}^{(p)}+m_{e})^{T}(\xi_{i}^{(p)}+m_{e})$

Step 6: Move along the inverse gradient direction $\Delta_{(i)}$ to estimate

$$\sum_{e(i+1)}=\sum_{e(i)}+\lambda\Delta_{(i)}$$

Where $\lambda>0$ is the step size (learning rate).

is converged , if it fulfills two conditions

1. Positive-definite,
2. The log-likelihood function increases: $l(\sum_{e(i+1)})>l(\sum_{e(i)})$.

Then the optimal correlation matrix is obtained at M^th^ final iteration

$\mathop{\rho_{e}}\limits^{\wedge} =B_{(M)}\sum_{e(M)}B_{(M)}$.

**APPENDIX C**

This Appendix provides the procedure of Markov Chain Monte Carlo method and is given as follows:

Step 1: First three-dimensional simulated regular rectangular grid is generated, and the grid nodes are initialized by assigning the random values drawn from the target histogram of each class.

Step 2: A 3-D template of the six-neighborhood (as shown in **Fig. S1**) structure is defined.

Step 3: Sample values at the known grid locations (conditioning data) are assigned and kept fixed throughout the entire simulation run.

Step 4: The conditioning data points are then transformed into indicator values.

Step 5: All grid node points (except conditioning data points) are revisited iteratively using Gibbs sampling until convergence is reached. The copula discriminant function defined earlier has been used for updating grid node values iteratively. For brevity, a detailed procedure of Gibbs sampling is not presented here.


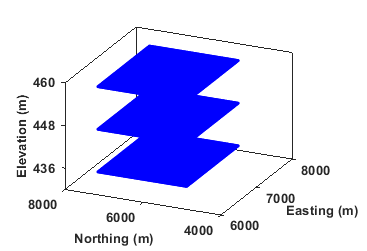


Template node

Grid node


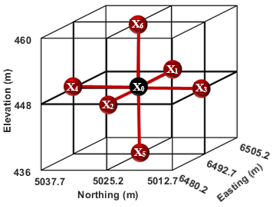


**Figure S1.** Shape of template geometry consisting of six neighboring nodes.

**References**

1. Duda R.O., Hart P.E. & Stork D.G. Pattern classification. 2nd edn. Wiley, New York (2001).
2. Coleman, T., Branch, M. A. & Grace, A. Optimization toolbox. For use with MATLAB*. User’s guide for MATLAB*, *5* (1999).
3. Hernández L., Tejero J. & Vinuesa J. Maximum Likelihood Estimation of the correlation parameters for elliptical copulas. arXiv preprint arXiv:1412.6316 (2014).
